# Nosocomial sepsis and drug susceptibility pattern among patients admitted to adult intensive care unit of Ayder Comprehensive Specialized Hospital, Northern Ethiopia

**DOI:** 10.1186/s12879-021-06527-4

**Published:** 2021-08-17

**Authors:** Tsega Cherkos Dawit, Reiye Esayas Mengesha, Mohamedawel Mohamedniguss Ebrahim, Mengistu Hagazi Tequare, Hiluf Ebuy Abraha

**Affiliations:** College of Health Sciences, Mekelle University, Mekele, Ethiopia

**Keywords:** Drug susceptibility, Intensive care unit, Mortality, Nosocomial sepsis

## Abstract

**Objective:**

Developing nosocomial sepsis within intensive care unit (ICU) is associated with increased mortality, morbidity, and length of hospital stay. But information is scarce regarding nosocomial sepsis in intensive care units of Northern Ethiopia. Hence, this study aims to determine the incidence of nosocomial sepsis, associated factors, bacteriological profile, drug susceptibility pattern, and outcome among patients admitted to the adult ICU of Ayder Comprehensive Specialized Hospital (ACSH), which is the largest tertiary hospital in Northern Ethiopia.

**Method:**

Facility-based longitudinal study was conducted by following 278 patients who were admitted for more than 48 h to adult ICU of ACSH, from October 2016 to October 2017. Data were collected from charts, electronic medical records, and microbiology registration book using a checklist. The collected data were subjected to descriptive statistics and multivariable logistic regression using SPSS version 25. Statistical significance was declared at p < 0.05.

**Result:**

Of all the patients, 60 (21.6%) of them acquired nosocomial sepsis. The risk of mortality was about two times higher among adult ICU patients who acquired nosocomial sepsis (RR = 2.2; 95% CI of RR = 1.3–3.5; p = 0.003). The odds of acquiring nosocomial sepsis among those who were on a mechanical ventilator (MV) and stayed more than a week were 5.7 and 9.3 times higher, respectively, than their corresponding counterparts. Among 48 isolates, Klebsiella was the most common pathogen. The isolates had a broad antibiotic resistance pattern for cephalosporins, penicillins, and methicillin.

**Conclusion:**

The incidence of nosocomial sepsis in the adult ICU patients of ACSH was higher when compared to the incidence reported from some African and Asian countries. Mortality was higher among patients who acquired nosocomial sepsis. Use of MV and longer length of ICU stay were the significant predictors of nosocomial sepsis. The isolates were resistant to several antibiotics. Therefore, strict application of infection prevention strategies and appropriate use of antibiotics is so crucial. As well, priority should be given to patients who develop nosocomial sepsis in ICU.

## Background

Sepsis is defined as systemic inflammatory response manifested by either hyperthermia or hypothermia, tachypnoea, leukocytosis, or leukopenia in the presence of proven or presumed infection [1]. Nosocomial sepsis is also defined as sepsis developing after 48 h of patient hospitalization [2]. Though it varies with geography and population characteristics, the most common pathogenic gram-positive bacteria causing nosocomial sepsis in intensive care unit (ICU) is *Staphylococcus aureus*, while organisms such as *Klebsiella*, *Acinetobacter*, *Pseudomonas*, and *Escherichia coli* species constitute the most common gram-negative species [3–7].

Developing nosocomial sepsis is common in critical care units and its prevalence can range from 9.6 to 17.7% [7–9]. Factors that predispose to the development of nosocomial sepsis in adult ICU patients are immunosuppression, prolonged hospitalization, intensive use of various equipment (e.g., catheter and mechanical ventilator (MV)), and old age [1, 8, 9]. The most common type of infections in the ICU are ventilator-associated pneumonia (VAP), central line-associated bloodstream infection (CLABSI), urinary catheter-related infection, and surgical site infection (SSI) [8–10]. Developing nosocomial sepsis within ICU is associated with increased mortality, morbidity, and length of hospital stay [7, 8].

In Ethiopia, the incidence of nosocomial sepsis is reported to be higher in intensive care units than inpatient wards [11, 12]. Evidence show that the prevalence of nosocomial sepsis in Ethiopia ranges from 26 to 36% [12, 13]. Studies made in teaching hospitals of Ethiopia also have identified older age, immunocompromising conditions, being on MV, chest tube, central-line catheters, and surgery as factors that increase the odds of hospital-acquired infection (HAI) [11, 14, 15]. At hospital level, a study done in the Amhara regional state of Ethiopia reported *Klebsiella* species and *Staphylococcus aureus* as the most commonly isolated HAI-causing pathogens [15]. But in surgical ward and surgical ICU, *Pseudomonas aeruginosa* and *Escherichia coli* are the major causes of HAI [13].

As a result, in Ethiopia, there are inconsistencies regarding the burden of nosocomial sepsis and the type of isolates causing nosocomial sepsis. Besides, these studies are not specific to adult ICU. Therefore, it is difficult to generalize their findings to adult ICU patients. Furthermore, some of the studies are outdated and information regarding antibiotic resistance is limited. Particularly, there is no such information in the Tigray regional state of Ethiopia. Hence, this study aimed to determine the incidence of nosocomial sepsis, associated factors, bacteriological profile, drug susceptibility pattern, and outcome among patients admitted to the adult ICU of Ayder Comprehensive Specialized Hospital (ACSH) which is the largest tertiary hospital in Tigray, Northern Ethiopia.

## Methods and materials

### Study area, design, and period

Ayder Comprehensive Specialized Hospital is located in Mekelle city and started to provide referral and non-referral clinical services in 2008 to an estimated 8 million population in its catchment areas of Tigray, Afar, and South-eastern parts of the Amhara Regional States. It provides a broad range of medical services to all age groups. Of the services provided, adult ICU holds a role in managing critically ill patients who require at most care and life supportive machines. It is equipped with MVs, defibrillators, perfusers, and other gadgets [16]. Health facility-based longitudinal study was conducted among patients admitted to adult ICU by following them throughout their ICU stay, from October 2016 to October 2017.

### Study population

All patients who stayed more than 48 h after admission to adult ICU at ACSH during the study period.

### Sample size

For this study, we included all (n = 294) patients who were admitted to adult ICU for more than 48 h during the study period.

### Data collection, source, and quality assurance

A checklist was developed and used to extract sociodemographic, clinical, and microbiological data. Data were collected from charts, electronic medical records, and microbiology registration books. Data were collected by trained internal medicine residents under the supervision of an internist.

### Study variables

Our outcome variables were nosocomial sepsis, mortality, bacteriological profile, and drug susceptibility. We defined nosocomial sepsis as sepsis developing after 48 h of ICU admission and it was dichotomized as “Yes” and “No”. Age, sex, residence, length of hospital stay, MV use, and central-line use were the independent variables.

### Body fluid sample collection and antimicrobial susceptibility testing

Under aseptic technique, body fluids (i.e., blood, urine, and tracheal secretion) were collected and transported to microbiology unit. Semiquantitative culture technique was used for urine samples. After a loop full of well-mixed and uncentrifuged urine and tracheal aspirate samples were inoculated onto Blood agar (BA) and MacConkey agar (MA), they were aerobically incubated at 37 °C for 24 h. Regarding blood samples, they were initially enriched on Brain Heart Infusion broth for 48 h and subsequently cultured on both MA and BA every 24 h for 3 days.

Next to the isolation of pathogens with colony characteristics, gram staining was performed to categorize isolates as gram positive and gram negative. Catalase, oxidase, coagulase, and optochin sensitivity tests were carried out for further identification of Gram positives. Gram negatives were also further identified using catalase, oxidase, motility, H_2_S and indole production, citrate utilization, triple sugar iron utilization, urea hydrolysis, and other biochemical tests.

Antimicrobial susceptibility of all isolates was determined by Kirby–Bauer disk diffusion method according to Clinical Laboratory Standard Institute (CLSI) [17]. For gram positive bacteria, the following discs were included: Penicillin G, Ampicillin, Erythromycin, Methicillin, Vancomycin, Ciprofloxacin, Chloramphenicol, Ceftriaxone, Sulphamethaxazole/trimethoprim, Meropenem, and Tetracycline. For gram negatives, Ampicillin, Amoxicillin/Clavulanic acid, Ciprofloxacin, Tetracycline, Gentamycin, Sulphamethaxazole/trimethoprim, Chloramphenicol, Ceftriaxone, Ceftazidime, Meropenem, Cephalothin, Amikacin, Piperacillin, and Tobramycin were used. After measuring and interpreting zone of inhibition using standard chart, the bacteria were labeled as susceptible (S), intermediate (I), or resistant (R).

### Data analysis

Data analyses were carried out using SPSS version 25. Frequency and percentage were employed to summarize categorical variables. Continuous variables were also described using an appropriate combination of measure of central tendency and measure of dispersion. Relative risk with its 95% confidence interval was calculated to quantify the effect of acquiring nosocomial sepsis on mortality. In addition, association between categorical variables was evaluated using Chi-square test of independence.

The relationship between the acquisition of nosocomial sepsis and its predictors was analyzed using binary logistic regression. Variables with a p-value < 0.25 in the univariable analysis were selected in the final multivariable logistic regression model. A p-value < 0.05 was considered to declare a statistical significance. The final multivariable model was checked for the goodness of fit and multicollinearity using Hosmer–Lemeshow test and variance inflation factor (VIF), respectively.

### Ethical approval

Ethical approval of the research was obtained from the Institutional Review Board (IRB) of Mekelle University, College of Health sciences. Permission to collect data was obtained from the chief clinical director’s office of ACSH. De-identification was applied by removing names and medical record numbers and replaced with codes.

## Results

**Fig. 1:**
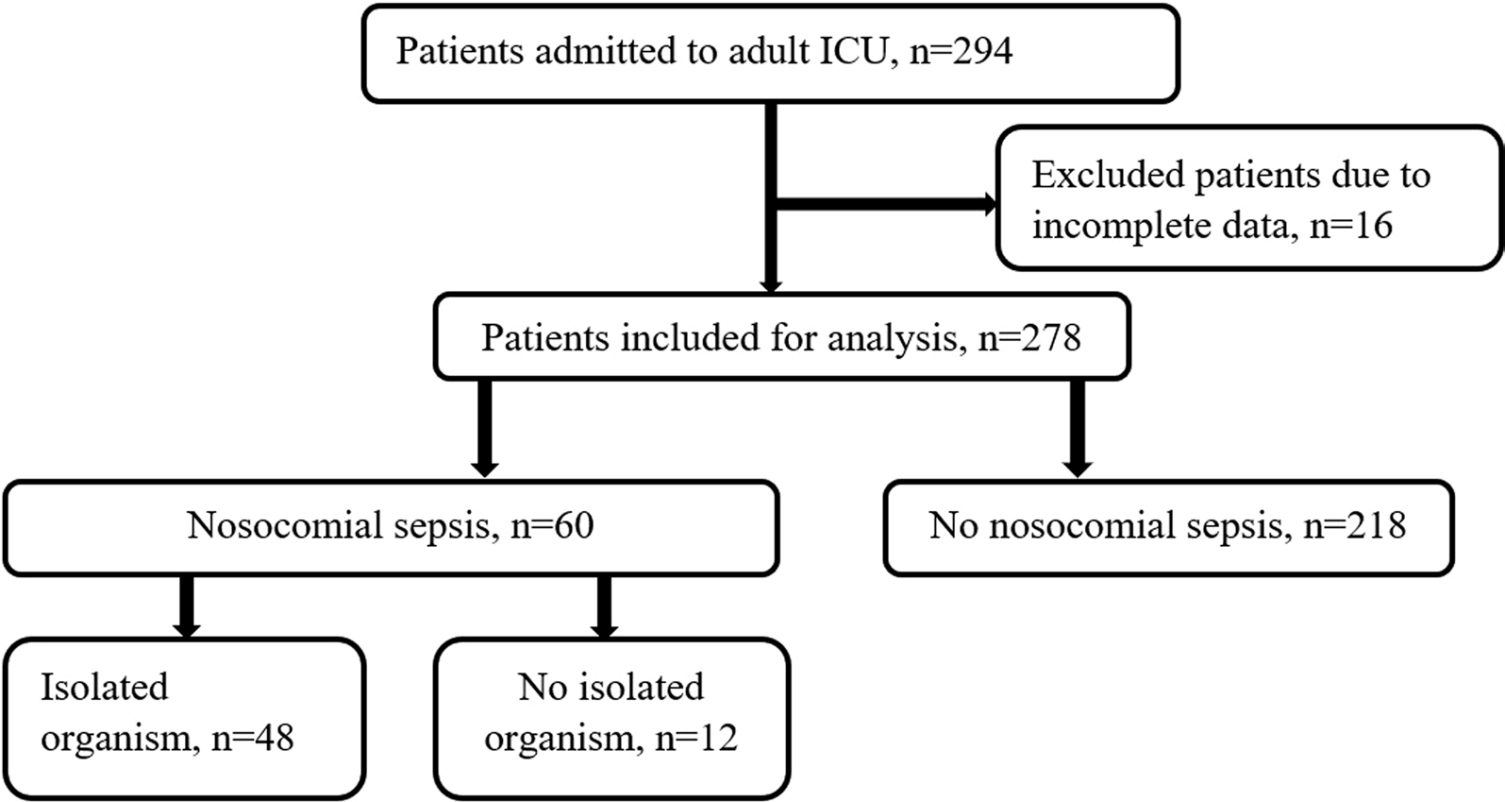
Flow diagram of patient recruitment, nosocomial sepsis, and isolated organisms

A total of 294 patients were admitted to the Adult ICU for more than 48 h during the study period. Of these, 278/294 (94.6%) patients with complete data were included. Sixty (21.6%) patients developed nosocomial sepsis and 48/60 (80%) of them had an isolated organism (Fig. 1).

### Sociodemographic and clinical characteristics

The median age of the participants was 42 (IQR = 31) years. Out of the patients included in the study, 156 (56.1%) were male and 163 (58.6%) were from an urban area (Table 1).

**Table 1:** Sociodemographic and clinical characteristics of the study participants adult ICU, ACSH, 2017 (n = 278)

Variables	Frequency	Percentage (95% CI)
Age in years		
< 40	123	44.2 (38.5–50.1)
40–64	120	43.2 (37.4–49.1)
65+	35	12.6 (9.2–17.1)
Sex		
Female	122	43.9 (38.1–49.8)
Male	156	56.1 (50.2–61.9)
Residence		
Urban	163	58.6 (52.7–64.3)
Rural	115	41.4 (35.7–47.3)
Mechanical ventilator use		
Yes	89	32.0 (26.8–37.8)
No	189	68.0 (62.2–73.2)
Central line use		
Yes	25	9.0 (6.1–13.0)
No	253	91.0 (87.0–93.9)
Urinary catheterization		
Yes	259	93.2 (89.5–95.6)
No	19	6.8 (4.4–10.5)
Length of stay (days)		
2–7 days	188	67.6 (61.9–72.9)
> 7	90	32.4 (27.1–38.1)
Nosocomial sepsis		
Yes	60	21.6 (17.1–26.8)
No	218	78.4 (73.2–82.9)
Focus of infection (n = 60)		
Respiratory	47	78.3 (65.8–87.2)
Urinary	10	16.7 (9.0–28.7)
Thrombophlebitis	3	5.0 (1.6–14.8)
Overall outcome		
Died	51	18.3 (14.2–23.4)
Discharged	227	81.7 (76.6–85.8)
Nosocomial sepsis outcome (n = 60)		
Died	19	31.6 (20.9–44.8)
Discharged	41	68.3 (55.2–79.1)

*ACSH* Ayder Comprehensive Specialized Hospital, *ICU* intensive care unit

Among the reasons for ICU admission, cardiovascular, neurologic, and respiratory conditions accounted for 88 (31.7%), 65 (23.4%), and 44 (14.4%), respectively. Of the cardiovascular conditions, acute coronary syndrome accounted for 53/88 (60.2%). Concerning neurologic conditions, head injury, stroke, and Guillain–Barre syndrome accounted for 29/65 (44.6%), 15/65 (23.1%) and 12/65 (18.5%), correspondingly. Pulmonary thromboembolism contributed for 13/44 (29.5%) of the respiratory admissions. Regarding admissions due to infectious conditions, tetanus and severe malaria accounted for 32% and 16% of cases, respectively. The remaining admission conditions include endocrine, gastrointestinal diseases, poisoning, electrolyte derangement, and intra-operative complications.

Nearly one-third of the study participants were on MV and only 9.0% of them required central-line use. For two-third of the patients, length of ICU stay was 1 week or less. Regarding urinary catheterization, all patients were catheterized, except 19 (6.8%) (Table 1). Among the non-catheterized patients, no individual developed nosocomial sepsis. In other words, all those who developed nosocomial sepsis were catheterized and there was a significant association between catheterization and nosocomial sepsis (χ^2^ (*N* = 278, *df* = 1) = 5.6, *p* = 0.018).

### Nosocomial sepsis and mortality

Of all the patients, 60 (21.6%) acquired nosocomial sepsis. For the patients with nosocomial sepsis, the main focuses of infection were respiratory (78.3%) and urinary (16.7%) (Table 1). The risk of mortality was about two times higher among those who acquired nosocomial sepsis (*Risk ratio (RR*) = 2.2; *95% CI of RR* = 1.3–3.5; *p* = 0.003).

Based on multivariable logistic regression model, the variables MV use and length of ICU stay were significantly associated with the nosocomial sepsis. Thus, the odds of acquiring nosocomial sepsis among those who were on MV were 5.7 times (AOR (95% CI) 5.7 (2.6–12.7), *p* < 0.001) higher than their counterparts. Those who stayed more than a week in the ICU had about nine times higher odds of nosocomial sepsis acquisition (AOR (95% CI) 9.3 (4.3–20.4), *p* < 0.001). The final multivariable model was good fit for the data (Hosmer–Lemeshow test: *χ*^*2*^ = 10.97, *df* = 8, *p* = 0.204) and had no multicollinearity issue (max VIF = 1.2, mean VIF = 1.1). ROC curve analysis revealed that the final model had very good accuracy with the area under the curve of 0.873 (Table 2).

**Table 2:** Factors associated with the acquisition of nosocomial sepsis, adult ICU, ACSH, 2017 (n = 278)

Variables	COR (95% CI)	p-value	AOR (95% CI)	p-value
Age in years				
< 40	1		1	
40–64	0.7 (0.4–1.4)	0.333	1.3 (0.6–3.2)	0.505
65+	0.5 (0.2–1.4)	0.181	0.8 (0.2–3.3)	0.806
Sex				
Female	1		1	
Male	1.6 (0.9–2.9)	0.119	1.4 (0.6–2.9)	0.412
Residence				
Urban	1		1	
Rural	1.7 (0.9–3.0)	0.069	1.6 (0.8–3.4)	0.207
Mechanical ventilator use				
Yes	11.9 (6.1–23.2)	< 0.001	5.7 (2.6–12.7)	< 0.001
No	1		1	
Length of stay				
2–7 days	1		1	
> 7 days	16.8 (8.2–34.3)	< 0.001	9.3 (4.3–20.4)	< 0.001
Central line use				
Yes	1.5 (0.6–3.7)	0.416	1.1 (0.3–3.7)	0.910
No	1		1	

*ACSH* Ayder Comprehensive Specialized Hospital, *AOR* adjusted odds ratio, *COR* crude odds ratio, *ICU* intensive care unit

### Microbiological profile of isolated samples

**Fig. 2:**
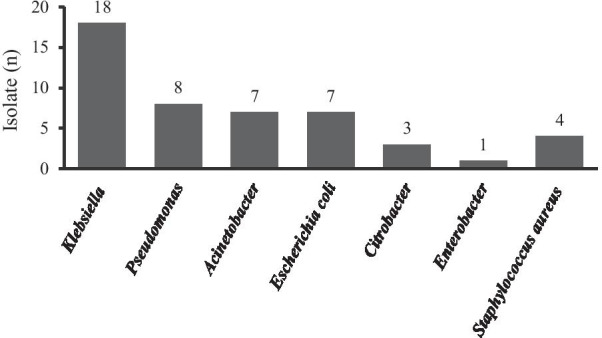
Isolated microorganisms from the septic patients in adult ICU, ACSH, 2017 (n = 48)

Samples were taken from blood, urine, and tracheal secretion of septic patients in the ICU. Of the samples taken, 48 isolates of organisms were found, of which 44/48 (91.7%) were gram-negative. The only gram-positive isolate was *Staphylococcus aureus* and accounted for 4/48 (8.3%). From the total isolates, *Klebsiella* was found to be the most common micro-organism identified, 18/48 (37.5%) (Fig. 2).

The gram-negatives had a broad antibiotic resistance pattern for cephalosporins, penicillins, and meropenem (Table 3). *Staphylococcus aureus* was also found to be resistant to penicillins and methicillin (Table 4).

**Table 3:** Distribution of antibiotics susceptibility pattern for isolated gram negative microbials from septic patients in Adult ICU, ACSH, 2017

Antibiotic	Sensitivity pattern	*Klebsiella* sensitivity % (isolates no)	*Pseudomonas* sensitivity % (isolates no)	*Escherichia coli* sensitivity % (isolates no)	*Acinetobacter* sensitivity % (isolates no)	*Citrobacter* sensitivity % (isolates no)
Ampicillin	R	100 (17/17)	100 (5/5)	89 (8/9)	100 (5/5)	100 (3/3)
	S	–	–	11 (1/9)	–	–
Penicillin	R	–	–	100 (1/1)	100 (1/1)	–
Tetracycline	R	90 (9/10)	100 (2/2)	83.3 (5/6)	100 (4/4)	100 (3/3)
	S	10	–	16.7	–	–
Doxycycline	R	66.7 (2/3)	100 (2/2)	100 (3/3)	66.7 (2/3)	100 (1/1)
	S	33.3	–	–	33.3	–
Chloramphenicol	R	71.4 (5/7)	100 (2/2)	66.7 (2/3)	100 (3/3)	100 (3/3)
	S	28.6	–	33.3	–	–
Ciprofloxacin	R	62.5 (10/16)	30()3/10)	77.8 (7/9)	83.3 (5/6)	100 (3/3)
	S	25	50	11.1	–	–
	I	12.5	20	11.1	16.7	–
Gentamycin	R	58.3 (7/12)	57.1 (4/7)	60 (3/5)	100 (3/3)	100 (3/3)
	S	16.7	14.3	20	–	–
	I	25	28.6	20	–	–
Cotrimoxazole	R	92.3 (12/13)	100 (2/2)	75 (3/4)	100 (3/3)	100 (3/3)
Augmentin	R	100 (13/13)	100 (2/2)	100 (4/4)	100 (4/4)	100 (3/3)
Amikacin	R	60 (3/5)	25 (2/8)	100 (1/1)	100 (3/3)	100 (2/2)
	S	20	37.5 (3/8)	–	–	–
	I	20	37.5 (3/8)	–	–	–
Cephalothin	R	100 (12/12)	100 (1/1)	100 (4/4)	100 (2/2)	100 (1/1)
Piperacillin	R	100 (1/1)	25 (1/3)	100 (1/1)	100 (2/2)	100 (1/1)
Ceftriaxone	R	100 (8/8)		100 (1/1)	100 (4/4)	100 (1/1)
	S	–	100 (1/1)			–
Ceftazidime	R	100 (9/9)	42.9 (3/7)	100 (1/1)	100 (5/5)	100 (1/1)
	S	–	57.1	–		–
Meropenem	R	10 (1/10)	50 (2/2)	–	100 (4/4)	100 (1/1)
	S	70 (7/10)	50	100 (1/1)		–
	I	20	–	–	–	–
Tobramycin	R	66.7 (2/3)	42.8 (3/5)	–	–	–
	S	33.3	28.6	–	–	–
	I	–	28.6	–	–	–
Cefepime	R	100 (3/3)	–	–	100 (4/4)	–
	S	–	100 (3/3)		–	–

*R* resistant, *S* susceptible, *I* intermediate, *ACSH* Ayder Comprehensive Specialized Hospital, *ICU* intensive care unit

**Table 4:** Distribution of antibiotics susceptibility pattern for isolated *Staphylococcus* *aureus* from septic patients in adult ICU, ACSH, 2017

Antibiotic	Sensitivity pattern	*Staphylococcus aureus* sensitivity % (isolates no)
Penicillin	R	100 (3/3)
Tetracycline	S	100 (1/1)
Doxycycline	R	33.3 (1/3)
	S	66.7 (2/3)
Chloramphenicol	R	100 (1/1)
Ciprofloxacin	R	100 (1/1)
Gentamycin	R	100 (2/2)
Cotrimoxazole	R	100 (1/1)
Ceftriaxone	R	100 (2/2)
Erythromycin	R	100 (2/2)
Vancomycin	R	–
	S	66.7 (2/3)
	I	33.3 (1/3)
Methicillin	R	100 (2/2)

*R* resistant, *S* susceptible, *I* intermediate, *ACSH* Ayder Comprehensive Specialized Hospital, *ICU* intensive care unit

## Discussion

Our study aimed at assessing the prevalence of nosocomial sepsis, associated factors, bacteriological profile, drug susceptibility pattern, and outcome of patients admitted to adult ICU of ACSH. The cumulative incidence of nosocomial sepsis in this study was 21.6%. MV use and length of ICU stay were independent predictors of nosocomial sepsis. Mortality was remarkably higher among those who developed nosocomial sepsis. The isolated microorganisms had a broad antibiotic resistance pattern for cephalosporins, penicillins, and meropenem.

The incidence of nosocomial sepsis in our setup was higher compared to studies done in Nigeria (15%) [8] and India (9.6–17.7%) [7, 9, 10]. Nosocomial sepsis happens due to inadequate hand hygiene technique, infrequent urinary catheter change, inadequately cleaned ventilator, lack of programmed ICU environment decontamination procedure, and lack of strict attendant visit protocol which make patients susceptible for various infections [18].

Those who were on MV had a higher risk (5.7-fold) of nosocomial sepsis. This finding was in tune with studies done in India [9, 10]. Additionally, those who stayed more than a week in the ICU had about nine times higher odds of nosocomial sepsis. This higher risk of nosocomial sepsis among those who stayed more than a week could be because of prolonged exposer to MV use. In our study, the use of MV was 64.4% in those who stayed more than a week in contrast to those who stayed a week and less, which was 16.5%. This difference was statistically significant (*χ*^*2*^ = 64.3, *df* = 1 and *p* < 0.001). Furthermore, longer ICU stay and MV use are thought to increase the risk of acquiring antibiotic-resistant infections [18, 19].

The overall mortality of ICU patients was 18.3%. But this mortality was significantly higher among those who acquired nosocomial sepsis (31.6%). Similar results regarding the mortality of patients with nosocomial sepsis are reported from studies done in Asia, Europe, and North America [10, 20–23]. In our study, the presence of nosocomial sepsis contributed considerably to poor outcome by increasing mortality risk significantly. Moreover, studies report that the burden of mortality due to nosocomial sepsis is higher in patients with organ dysfunction and those who had surgery on an emergency basis [21, 23].

Among the body fluid samples taken, 48 isolates of organisms were found, of which 91.6% were gram-negative and 8.3% were gram-positive and this finding was consistent with studies carried out in Nigeria, India, and Europe [8, 9, 20, 24]. *Klebsiella* was found to be the most common micro-organism followed by *pseudomonas*, *Escherichia coli*, and *Acinetobacter*. This is parallel with the studies of Nigeria, India and Fiji [8, 20, 24]. For instance, in Fiji’s study, the commonest pathogens isolated were *Klebsiella pneumoniae*, *Acinetobacter*, and *Pseudomonas* [20].

*Klebsiella* was resistant to ampicillin, ceftazidime, and cefepime in all tested isolates and showed a broad resistance pattern to other antibiotics, but was found to be sensitive to meropenem in 66.7%. *Pseudomonas* had a sensitivity of 57.1% and 50% to ceftazidime and meropenem respectively. Higher resistance to meropenem was found in Acinetobacter, where all 4 isolates were resistant to the antibiotic. The isolated *Staphylococcus aureus* was also methicillin-resistant but vancomycin sensitive. These results indicate that there is a broad resistance to penicillin, cephalosporins, and other antibiotics in our adult ICU. Such kind of broad resistance is reported by several studies done in ICU of different countries [25–36].

The use of antibiotics to patients should be evidence-based during a highly suspected infection and bacteriologic evidence of infection. Frequent use of antibiotics in patients with or without sepsis renders most organisms to be antibiotic-resistant. When indicated, the use of antibiotics should be guided with a bacteriological profile. Providing drugs to a patient harboring resistant microorganisms has negative impacts including financial burden, longer hospital stay, deterioration, and death of the patient (18, 19).

## Conclusion

There is a higher incidence of sepsis in our adult ICU compared to the incidence reported from some African and Asian countries. Use of MV and longer length of in-hospital stay were significant risk factors for nosocomial sepsis. Furthermore, nosocomial septicaemia had a significant effect on mortality. The identified organism were mainly gram negatives.

## Limitations of the study

Body fluid culture was not done for all patients. In addition, antibiotic sensitivity test was inconsistent across each group of organisms (i.e., gram negative and gram positive).

## Recommendation

Great emphasis should be given to the high incidence of nosocomial sepsis. Setting up motivated and persistent infection prevention techniques is required. This includes developing strict protocols and practice which is adherent to the protocol. These include adequate cleaning of mechanical ventilators and other devices before and after use in a patient. The use of antibiotics should be guided with appropriate clinical sepsis diagnosis and basing the selection on the bacteriological profile of the patient. As well, hospital management and policymakers like the ministry of health should give priority to this higher incidence of nosocomial sepsis in ICU and reinforce existing infection prevention and control strategies of Ethiopia. Besides, nosocomial sepsis management guidelines need to be developed based on available published and unpublished local research works and continuous nosocomial sepsis surveillance should be considered to assess the trend.

## Data Availability

The datasets used and/or analysed during the current study available from the corresponding author on reasonable request.

## Abbreviations

ACSH: Ayder Comprehensive Specialized Hospital
AOR: Adjusted odds ratio
BA: Blood agar
CLABSI: Central line-associated bloodstream infection
CLSI: Clinical Laboratory Standard Institute
COR: Crude odds ratio
HAI: Hospital acquired infection
ICU: Intensive care unit
IQR: Inter-quartile range
IRB: Institutional Review Board
MA: MacConkey agar
MV: Mechanical ventilator
ROC: Receiver operating characteristic
RR: Risk ratio
SSI: Surgical site infection
VAP: Ventilator-associated pneumonia
VIF: Variance inflation factor
